# Convergence study of the *h*-adaptive PUM and the *hp*-adaptive FEM applied to eigenvalue problems in quantum mechanics

**DOI:** 10.1186/s40323-017-0093-0

**Published:** 2017-12-12

**Authors:** Denis Davydov, Tymofiy Gerasimov, Jean-Paul Pelteret, Paul Steinmann

**Affiliations:** 1 Chair of Applied Mechanics, University of Erlangen-Nuremberg, Egerlandstr. 5, 91058 Erlangen, Germany; 2 Institute of Applied Mechanics, Technische Universitaet Braunschweig, Bienroder Weg 87, 38106 Braunschweig, Germany

**Keywords:** Adaptive finite element method, Partition-of-unity method, Error estimators, Schrödinger equation, Local interpolation error estimates, Density functional theory

## Abstract

In this paper the *h*-adaptive partition-of-unity method and the *h*- and *hp*-adaptive finite element method are applied to eigenvalue problems arising in quantum mechanics, namely, the Schrödinger equation with Coulomb and harmonic potentials, and the all-electron Kohn–Sham density functional theory. The partition-of-unity method is equipped with an a posteriori error estimator, thus enabling implementation of error-controlled *adaptive* mesh refinement strategies. To that end, local interpolation error estimates are derived for the partition-of-unity method enriched with a class of exponential functions. The efficiency of the *h*-adaptive partition-of-unity method is compared to the *h*- and *hp*-adaptive finite element method. The latter is implemented by adopting the analyticity estimate from Legendre coefficients. An extension of this approach to multiple solution vectors is proposed. Numerical results confirm the theoretically predicted convergence rates and remarkable accuracy of the *h*-adaptive partition-of-unity approach. Implementational details of the partition-of-unity method related to enforcing continuity with hanging nodes are discussed.

## Introduction

Recently there has been an increase of interest in applying finite element (FE) methods to partial differential equations (PDEs) in quantum mechanics [1–14]. In order to improve the accuracy of the solution, the basis set can be adaptively expanded through either refinement of the mesh (*h*-adaptivity) or the basis functions can be augmented by the introduction of higher polynomial degree basis functions (*p*-adaptivity). Since the solution is not smooth and contains cusp singularities, the application of the *h*-adaptive FEM may require very fine meshes and could be computationally inefficient. There are several approaches to circumvent this problem.

From the physical point of view, for ab initio calculation of molecules often core electrons (as opposed to valence electrons) behave in a similar way to single atom solutions. Thus one possesses an a priori knowledge of a part of the solution vectors to the eigenvalue problem. One of the approaches used to introduce this into a FE formulation is the partition-of-unity method (PUM) [15–17], which is a generalization of the classical FE method. In PUM the enrichment functions are introduced into a basis as products with standard FE shape functions, thereby enlarging the standard FE space. As the standard FE functions satisfy the partition-of-unity property (that is, they sum to one in the whole domain), the resulting basis can reproduce enrichment functions exactly. For an overview on PUM applied to continuum mechanics we refer the reader to [18–20].

An alternative approach to the above is to combine *h*- and *p*-adaptivity resulting in what is termed as *hp*-adaptive FEM. For an overview of *hp*-adaptive refinement strategies we refer the reader to [21]. The general idea is that when the exact solution is smooth on the given element, *p*-adaptive refinement is more efficient and leads to a faster convergence with respect to the number of degrees of freedom; whereas if the solution is non-smooth (singular), *h*-adaptive refinement is performed. Thus in addition to a reliable error estimate and the choice of the marking strategy of elements for refinement, *hp*-adaptive methods need to decide which type of refinement to perform on a given element. In this work we use methods based on smoothness estimation [22–27]. As those methods are normally employed for problems with a single solution vector, we propose an extension to multiple solution vectors as is required for the here considered eigenvalue problems.

Herein, our main focus is application of *h*-adaptive PUM to PDEs in quantum mechanics, namely to the Schrödinger equation and the all-electron density functional theory (DFT) [28, 29]. Application of the PUM to the above problems holds a significant promise to improve on accuracy of a standard (non-enriched) FE approximation. The corresponding numerical evidence can be found in [9], where convergence studies for PUM solutions obtained on *uniformly* refined meshes are performed.

The novelty of our paper is that the PUM will be equipped with an a posteriori error estimator, thus enabling implementation of error-controlled *adaptive* mesh refinement strategies. Derivation and implementation of the PUM in computational solid mechanics is nowadays very well-acknowledged and established area of research, yet the authors are not aware of any other work which applies the *h*-adaptive PUM to DFT.

We will also compare the PUM to *hp*-adaptive FEM in terms of the efficiency with respect to the number of degrees of freedom. Although there are publications on the topic of *hp*-adaptive FEM applied to DFT [1], they lack any numerical studies and are limited to a pre-defined refinement strategy of hexahedra that admit nuclei only at its vertices. In order to apply the *hp*-adaptive FEM to DFT, in this paper we propose an extension of the smoothness estimate approach using Legendre coefficients [22–25] to multiple solutions vectors.

The outline of this paper is as follows: In the section on “Theory”, we introduce the eigenvalue problem studied here. The PUM and error estimators are also discussed. We also explain the strategy employed to decide between *h*- and *p*-adaptive refinement for the *hp*-adaptive FEM. Results of numerical studies of the chosen systems are presented in section titled “Results and discussion”, followed by some conclusions. In Appendix A we rigorously derive the local interpolation error estimates for enrichment with a class of exponential functions; Appendix B describes the approach applied to solve single atom DFT in radial coordinates within application of the PUM; Appendix C discusses implementational details of PUM within the context of the deal.II [30] library.

## Theory

In quantum mechanics we seek the *N* lowest eigenpairs $(\lambda _\alpha ,\psi _\alpha )$ of the Schrödinger equation [31]

1 $$\begin{aligned} \Bigg [-\frac{1}{2} \nabla ^2 + V(\mathbf{x }) \Bigg ]\, \psi _\alpha (\mathbf{x })&= \lambda _\alpha \psi _\alpha (\mathbf{x }) \quad \mathrm{on}\; \Omega \,, \nonumber \\ \psi _\alpha (\mathbf{x })&= 0 \quad \mathrm{on}\; \partial \Omega , \\ \int _\Omega \psi _\alpha (\mathbf{x }) \psi _\beta (\mathbf{x }) \mathrm{d} \mathbf{x }&= \delta _{\alpha \beta } \,.\nonumber \end{aligned}$$

Here $\alpha$ is the index of the eigenpair, $\Omega$ is a Lipschitz domain^1^ in $\mathbb {R}^3$ and $\delta _{\alpha \beta }$ is the Kronecker delta . In Kohn–Sham all-electron density functional theory [28, 29], the potential $V(\mathbf{x })$ depends on eigenvectors thus rendering the problem nonlinear. For a molecular system consisting of $N_e$ electrons and M nuclei of charges $\{Z_\mathrm{I}\}$ located at the (fixed) positions $\{{\mathbf {\mathsf{{R}}}}_\mathrm{I}\}$, the ground state electron density $\rho (\mathbf{x }):= \sum _{\alpha =1}^{N} f_\alpha \left|\psi _{\alpha }(\mathbf{x }) \right|^2$ can be obtained by finding the *N* lowest eigenpairs of Eq. (1). Here $f_\alpha$ is the partial occupancy number^2^ of the $\alpha$-orbital such that $\sum _{\alpha =1}^{N} f_\alpha = N_e$, $V=V_{\mathrm{ion}}+V_{Hartree}+V_{\mathrm{xc}}$ is composed of the ionic potential $V_{ion}=-\sum _{\mathrm{I=1}}^M \frac{Z_{\mathrm{I}}}{\left| \mathbf{x } - {\mathbf {\mathsf{{R}}}}_{\mathrm{I}} \right| }$, the Hartree potential $V_{Hartree}=\int _\Omega \rho (\mathbf{x }')/|\mathbf{x }- \mathbf{x }'|\mathrm{d} \mathbf{x }'$, and the (given) exchange-correlation potential $V_{xc}(\rho )$. As a result, the potential *V* depends on the density $\rho$ which is given in terms of eigenvectors $\{\psi _\alpha \}$, making the problem nonlinear. From practical perspective $V_{Hartree}$ electrostatic potential is obtained by solving the associated Laplace equation; together with (1) they are solved sequentially untill convergence in density fields $\rho$ is attained. For further details on the FE solution of DFT, we refer to our previous work [2] and literature cited therein.

The weak form of Eq. (1) reads^3^

2 $$\begin{aligned} \begin{aligned} \int _{\Omega } \Bigg [ \frac{1}{2} \nabla v \cdot \nabla \psi _{\alpha } + v V \psi _\alpha \Bigg ] \mathrm{d} \mathbf{x }&= \lambda _\alpha \int _{\Omega } v\psi _\alpha \mathrm{d} \mathbf{x } \quad \forall v \, \in H^1_0(\Omega )\,, \\ \int \psi _\alpha \psi _\beta \mathrm{d} \mathbf{x }&= \delta _{\alpha \beta } \,. \end{aligned} \end{aligned}$$

We then introduce a FE triangulation $\mathcal {P}^h$ of $\Omega$ and the associated FE space of continuous piecewise elements of a fixed polynomial degree : $\psi _\alpha \in V^h \subset H^1_0 (\Omega )$. The FE solution to the problem is then defined by

3 $$\begin{aligned} \begin{aligned} \int _{\Omega } \Bigg [ \frac{1}{2} \nabla v^h \cdot \nabla \psi _{\alpha }^h + v^h V \psi _\alpha ^h \Bigg ] \mathrm{d} \mathbf{x }&= \lambda _\alpha ^h \int _{\Omega } v^h \psi _\alpha ^h \mathrm{d} \mathbf{x } \quad \forall \, v^h \in V^h\,, \\ \int \psi _\alpha ^h \psi _\beta ^h \mathrm{d} \mathbf{x }&= \delta _{\alpha \beta } \,. \end{aligned} \end{aligned}$$

### Partition-of-unity method

The classical FEM with piecewise linear ansatz spaces requires very fine meshes for adequate accuracy when the solution is not smooth or is highly oscillatory; this increases the computational cost of solving the problem. The PUM proposed by Melenk and Babuska in [15, 16] can address this issue. The main feature of the PUM is the inclusion of an a priori knowledge about the solution properties into the FE space. The PUM enriches the vector space spanned by standard FE basis functions $N_i(\mathbf{x })$ (e.g. polynomials) by products of these functions with functions $f_{j}(\mathbf{x })$ that contain a-priori knowledge about the solution

4 $$\begin{aligned} \psi _\alpha ^h(\mathbf{x }) = \sum _{i \in I} N_i(\mathbf{x }) \,\left[ \psi ^i_\alpha + \sum _{j \in S} f_j(\mathbf{x }) \widetilde{\psi }^{ij}_\alpha \right] . \end{aligned}$$

Here $\psi ^i_\alpha$ are standard degrees-of-freedom (DoFs) and $\widetilde{\psi }^{ij}_\alpha$ are additional DoFs associated with the shape functions $N_i(\mathbf{x })$ and the enrichment functions $f_j(\mathbf{x })$; *I* is a set of all nodes and *S* is the set of enrichment functions. Since (possibly global) enrichment functions $f_j(\mathbf{x })$ are multiplied with $N_i(\mathbf{x })$ which has local support, the product also has local support and therefore matrices arising from the weak form remain sparse. Also, since the standard shape functions satisfy the partition of unity property $\sum _i N_i(\mathbf{x }) \equiv 1$, the resulting vector space can reproduce enrichment functions $f_j(\mathbf{x })$ exactly.

Note that (4) is a more general approach than enriching the basis with $f_j$ alone (i.e. without multiplying by $N_i$, as is employed in [32]). Granted the partition of unity property of $N_i$, this case can be obtained from (4) by requiring all DoFs associated with a given enrichment function $f_j$ to have the same value.

### Error estimator

A posteriori error estimation analysis for FE approximations of (second-order) eigenvalue problems has been a topic of intensive study within the last several decades, both from theoretical and implementational standpoints. We refer the interested reader to [13, 14, 33–39], where two “conventional” types of error estimators, namely residual- and averaging-based error estimators, are presented.

In general, a discretization error in approximated eigenfunctions, $\psi -\psi ^h$, measured in a suitable norm (e.g. $L^2$-norm and energy norm, induced by the bilinear form of a problem), as well as in approximated eigenvalues, $|\lambda -\lambda ^h|$, can be estimated from above. That is,

5 $$\begin{aligned} \left\| \psi -\psi ^h\right\| \le C_1\eta , \end{aligned}$$

and

6 $$\begin{aligned} |\lambda -\lambda ^h|\le C_2\eta ^2, \end{aligned}$$

where $C_1,C_2$ are the so-called stability constants that are independent of the mesh size and $\eta$ is the *explicitly computable* 4 error upper-bound, see e.g. [34, 38] for details. These equations are typically termed *(global) error estimators*. The bound $\eta$ reads as

$$\begin{aligned} \eta :=\left[ \sum _{K\in \mathcal {P}^h} \eta _K^2 \right] ^\frac{1}{2}, \end{aligned}$$

where summation is performed over all elements in $\mathcal {P}^h$ and $\eta _K$ is the *(local) error indicator*, a quantity showing a discretization error of $\{\psi ^h,\lambda ^h\}$ element-wise, that is, on every fixed *K*. With multiple solutions available (in this case, eigenpairs $\{\psi ^h_\alpha ,\lambda ^h_\alpha \}$), $\eta _K$ will be a sum of discretization errors of the corresponding eigenpairs on a given element *K*, that is

$$\begin{aligned} \eta _K:=\left[ \sum _{\alpha } \eta _{K,\alpha }^2 \right] ^\frac{1}{2}. \end{aligned}$$

For a standard (non-enriched) $\mathbb {Q}_1$-based finite element solution of (1), a local indicator $\eta _{K,\alpha }$ of *residual* type reads as follows (see [13, 14, 34, 35, 38, 39] for details):

7 $$\begin{aligned} \eta _{K,\alpha }^2:= & {} h_K^2 \int _K \left[ \,\Bigg ( -\frac{1}{2} \nabla ^2 + V(\mathbf x )\Bigg ) \psi ^h_\alpha - \lambda ^h_\alpha \,\psi ^h_\alpha \right] ^2 \mathrm{d} \mathbf{x } \nonumber \\&+ \, h_K \sum _{e\subset \partial K} \int _e \left[ \left[ -\frac{1}{2}\nabla \psi ^h_\alpha \cdot \mathbf{n }\right] \right] _e^2 \mathrm{d} \mathbf{a }, \end{aligned}$$

where $[[-\frac{1}{2}{\nabla } \psi ^h_\alpha \cdot \mathbf{n } ]]_e:=\left[ -\frac{1}{2} {\nabla } \psi ^h_\alpha \,|_{K}+\frac{1}{2}\nabla \psi ^h_\alpha \,|_{K'} \right] \cdot \mathbf{n }_e$ represents the jump of the gradient across interface *e* between two adjacent elements *K* and $K'$, $\mathbf{n }_e$ is the outward unit normal vector to *e* and $h_K:=\mathrm {diam}(K)$.

One of the key findings of our work is the proof that indicator (7) also holds (with no modification due to the enrichment usage) in the PUM with the exponential enrichment function $f(\mathbf{x }) = \exp {(-\mu \left|\mathbf{x } \right|^k)}$. In Appendix A, we derive and prove the related local interpolation error estimates required for the derivation of the error indicator (7).

### hp-adaptive solution

There have been numerous works devoted to *hp*-adaptive refinement [22–25, 40–42] including a comparison of different methods [21]. The main difficulty that *a posteriori* *hp*-adaptive methods aim to address is the following: once an error is estimated and a certain subset of elements is marked for refinement, one has to choose between *h*- or *p*-refinement for each element. In this work we adopt an *hp*-refinement method based on the estimate of the analyticity of the solution^5^ on the reference element via expansion into Legendre bases [22–25]. In particular, the FE solution is analytic on element *K* if, and only if, there exists constants $C_K$ and $\sigma _K$ such that

8 $$\begin{aligned} \left|a_{ijk}^K \right| \le C_K \exp (-\sigma _K [i+j+k]) , \end{aligned}$$

where $a_{ijk}$ are Legendre coefficients; see [25] for further details. We chose to estimate the decay coefficient $\sigma _K$ by performing a least squares fit of Legendre coefficients in each direction $\ln \left|a_{d,i}^K \right| \sim \ln C_K^d - \sigma _K^d \, i$ for $1 \le i \le p_K$, and then use the minimum decay coefficient as the final value $\sigma _K=\min _d \sigma _K^d$. When $\sigma _K$ value is below a chosen parameter $\sigma _0$, the solution is considered to be smooth at *K* and thus *p*-refinement is performed, otherwise *h*-refinement is executed. For initially linear FEs *p*-refinement is always performed. We note that methods based on the decay rate of the expansion coefficients were found in [21] to be the best choice as a general strategy for the *hp*-adaptive solution of elliptic problems. To the best of our knowledge there is, however, very little (numerical) study of those methods applied to DFT. The only paper we are aware of [1] lacks any numerical results. We also note that, in the majority of cases, *hp*-adaptive FEM is applied to problems in $\mathbb {R}^1$ and $\mathbb {R}^2$. Thus we also aim to evaluate how well the smoothness estimators proposed in the literature work for eigenvalue problems in $\mathbb {R}^3$ that are relevant to quantum mechanics. In order to extend this *hp*-refinement strategy to the eigenvalue problem, that is when there are multiple vectors represented using the same FE basis, we propose the following approach. For each element we find an eigenvector which contributes the most to the total element’s error. The smoothness of this vector is the basis on which we decide to perform *h*-refinement or *p*-refinement. The rationale behind this approach is that we aim at minimizing the error the most during a single refinement step while being conservative and avoiding performing both *h*- and *p*-refinement on the same element. We also investigated allowing both *h*- and *p*-adaptive refinement of a single cell based on smoothness estimation of all eigenvectors, but ultimately found that this procedure leads to qualitatively similar results for the problems studied herein.

Finally, for the error indicator we adopt the following expression [43]

9 $$\begin{aligned}&\eta ^2_{K,\alpha } := \frac{h_{K}^2}{p_K^2} \int _{K} \left[ \,\Bigg ( -\frac{1}{2} \nabla ^2 + V(\mathbf{x })\Bigg ) \psi ^h_\alpha - \lambda ^h_\alpha \,\psi ^h_\alpha \right] ^2 \mathrm{d} \mathbf{x }\nonumber \\&\quad + \sum _{e \subset \partial K} \frac{h_{e}}{2 p_e} \int _{e} \left[ \left[ -\frac{1}{2}{\nabla } \psi _\alpha ^h \cdot \mathbf{n } \right] \right] _e^2 \mathrm{d} \mathbf{a } \,, \end{aligned}$$

where $h_e$ is the face’s diameter, $p_K$ is the element’s polynomial degree and $p_e$ is the maximum polynomial degree over two elements *K* and $K'$ adjacent to the face *e*.

The derivation of the error estimators for *hp*-FEM usually requires the polynomial degree of neighbouring elements to be comparable, namely that there exists $\gamma$ and $\Gamma$ such that $\gamma p_K \le p_{K^\prime } \le \Gamma p_K$ for all elements $K, K^\prime$ that have a non-empty intersection. In order to reflect this assumption in our numerical scheme, we propose that an additional step which limits the differences in polynomial degrees among elements be performed. More precisely, after *hp*-adaptive refinement is executed, then for each element *K*, we find the maximum polynomial degree among its neighbouring elements $p_\mathrm{neigh}^\mathrm{max}$ and if $p_\mathrm{neigh}^\mathrm{max} > p_K + 2$ then we set $p_K \leftarrow p_K+1$.

## Results and discussion

If not explicitly stated otherwise, the results below are obtained for the following configuration: (i) the initial polynomial degree for non-enriched DoFs is one for *hp*-adaptive FEM; (ii) linear shape functions are used in PUM to introduce enrichments, higher order elements were not employed as the interpolation error estimates are derived only for linear elements and thus limit the applicability of the error indicator stated in Eq. (7) ; (iii) a Gaussian quadrature rule with $20^3$ points is used for enriched elements in the eigenvalue problem; (iv) the Dörfler marking strategy with $\theta = 0.6$ is used to mark elements for refinement; (v) Gauss–Legendre–Lobatto supports points are used for the *hp*-adaptive FEM basis to improve the condition number; (vi) in case of *hp*-adaptive refinement the highest polynomial degree is limited to 8 for computational efficiency reasons; (vii) the radius in which enriched FEs are employed is heuristically chosen for each numerical example; (viii) following [25] we choose $\sigma _0=1.0$ as a parameter in the smoothness estimator.^6^ Implementation details of the partition-of-unity method in deal.II [30] finite element library are given in Appendix C.

### Schrödinger equation

In this section we consider the Schrödinger equation Eq. (1) with two different (spherical) potentials $V(\mathbf{x })=V(\left|\mathbf{x } \right|)$.^7^ The first case is the Coulomb potential $V(\mathbf{x }) = -\,1/\left|\mathbf{x } \right|$, which corresponds to a Hydrogen atom. The eigenvalues of this problem are degenerate. In $\mathbb {R}^3$, on each energy level *n* there are $n^2$ eigenvalues $\lambda _n = \lambda _1/n^2$, where $\lambda _1 = -\,1/2$ [31]. The eigenfunction corresponding to the lowest eigenvalue reads

10 $$\begin{aligned} \psi _1(\mathbf{x }) = \frac{1}{\sqrt{\pi }} \exp \left( -\left|\mathbf{x } \right| \right) . \end{aligned}$$

The radial component of the eigenfunctions at the next energy level are $R_{2,0}=[1-\left|\mathbf{x } \right|/2]\exp (-\left|\mathbf{x } \right|/2)$ and $R_{2,1}=\left|\mathbf{x } \right|/2\exp (-\left|\mathbf{x } \right|/2)$.

The second potential we will consider is a harmonic potential $V(\mathbf{x })=\left|\mathbf{x } \right|^2/2$ that leads to a harmonic oscillator problem. The eigenvalues for this problem are also degenerate; in $\mathbb {R}^3$ they are given by $\lambda _n = n+1/2$ for *n*th energy level. The lowest two have a degeneracy of 1 and 3, respectively. The (unnormalized) eigenfunction corresponding to the lowest eigenvalue is

11 $$\begin{aligned} \psi _1(\mathbf{x }) = \exp \left( -\left|\mathbf{x } \right|^2/2 \right) . \end{aligned}$$

**Fig. 1:**
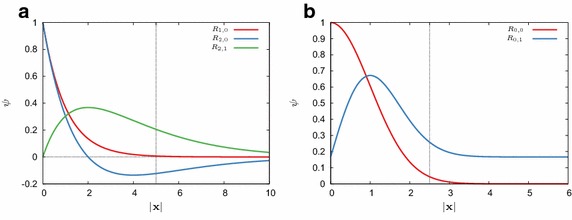
Radial components of eigenfunctions for different potentials *V*(**x**). The dotted vertical line indicates the smallest initial mesh size which will be used in our numerical calculations. **a** Coulomb. **b** Harmonic

The radial component of the next eigenfunction is $R_{0,1}(\mathbf{x }) = \left|\mathbf{x } \right| \exp \left( -\left|\mathbf{x } \right|^2/2 \right)$. Figure 1 shows radial components of eigenfunctions for the Coulomb and the harmonic potential. It is clear that in order to have a low interpolation error for a standard Lagrange FE basis, a very fine mesh will be required near the origin. For such non-smooth solutions we will see that by introducing enrichment functions the interpolation error of the resulting FE basis will be greatly reduced.

The initial mesh used to solve the Schrödinger equation is obtained from 3 global mesh refinements of the single element in $\Omega =[-\,20;20]^3$ for the Coulomb potential and $\Omega =[-\,10;10]^3$ for the harmonic potential. For the PUM only 8 elements adjacent to the singularity that is located at the origin are marked for enrichment.

**Fig. 2:**
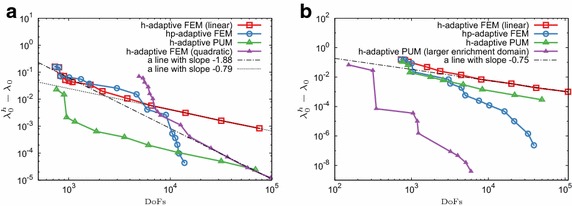
Error convergence rates for an eigenproblem with a single eigenpair. **a** Coulomb potential. **b** Harmonic potential

**Fig. 3:**
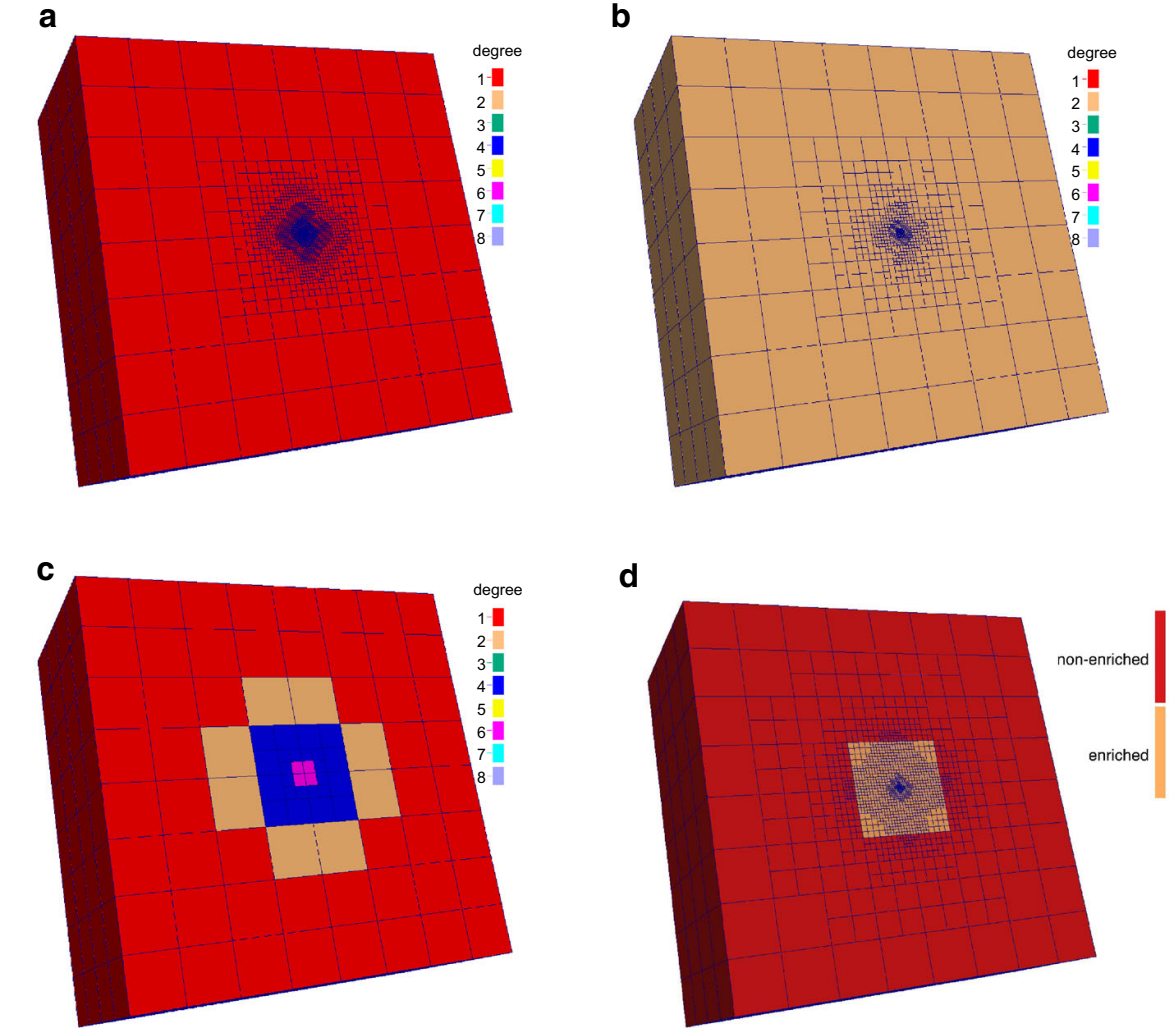
Cross-sections of the final adaptive meshes for the Coulomb potential when solving for a single eigenpair. **a** *h*-adaptive FEM (linear). **b** *h*-adaptive FEM (quadratic). **c** *hp*-adaptive FEM. **d** *h*-adaptive PUM (linear)

First, we examine the convergence in case when a single eigenpair is required in the Schrödinger equation with two different potentials. Figure 2 compares the *h*-adaptive FEM, *hp*-adaptive FEM and *h*-adaptive PUM, whereas Fig. 3 shows the cross-sections of meshes for the last refinement step.

For both combinations of potentials and enrichment functions, the *h*-adaptive PUM is superior to *h*-adaptive FEM. In particular, for the last refinement step the PUM solution is about two orders more accurate than the *h*-adaptive FEM with the same number of DoFs in the case of the Coulomb potential. For the harmonic potential this value is smaller. The asymptotic convergence rate of the *h*-adaptive PUM with the default enrichment radius is very similar to that of the *h*-adaptive FEM for both problems (compare green and red lines in Fig.  2), which supports our theoretical findings.

The advantage of the *h*-adaptive PUM also depends on the enrichment radius with respect to the underlying exact solution. To examine this effect we employ an initial mesh obtained only by two global refinements of a single element and mark the 8 elements adjacent to the origin for enrichment. With this approach we effectively consider a larger enrichment domain $[-\,5;5]^3$ instead of $[-\,2.5;2.5]^3$. Importantly, the numerically non-zero part of the underlying analytical solution will be almost fully contained in those 8 elements (see Fig. 1b). From the numerical results we observe that for the most refined stage the *h*-adaptive PUM displays an error which is about 6 orders of magnitude less than the same method with the smaller enrichment domain (compare purple and green lines in Fig. 2b).

For the case of a single eigenpair, the *hp*-adaptive FEM performs remarkably well and, unless a larger enrichment radius is used in *h*-PUM, it converges to the higher tolerance with fewer number of DoFs (compare blue and green lines in Fig. 2).

**Fig. 4:**
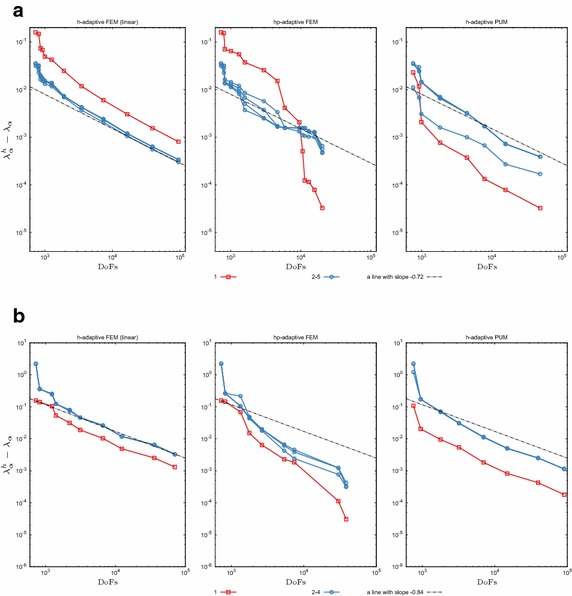
Convergence of eigenvalues from the first two energy levels for the Schrödinger equation in the course of adaptive refinement. Red lines denote the lowest eigenvalue, whereas blue lines correspond to degenerate eigenvalues on the next energy level. **a** Coulomb potential (4 out of 5 eigenvalues are degenerate). **b** Harmonic potential (3 out of 4 eigenvalues are degenerate)

Now let us turn our attention to a more realistic scenario where one seeks multiple eigenpairs whereby an *a priori* knowledge is available only for the first eigenfunction. Figure 4 plots the convergence of the first 5 eigenvalues for the Coulomb potential and the first 4 eigenvalues for the harmonic potential for the different methods. For both problems the *h* adaptive PUM again has remarkable convergence properties, superior to *h*-adaptive FEM. It is important to note that even though in the PUM the enrichment function corresponds to the first eigenfunction only, other eigenpairs in the case of the harmonic potential tend to converge faster than the standard *h*-adaptive FEM case, as can be observed in Fig. 4b. The same applies to the spherical orbital at the second energy level of the Hydrogen atom; see Fig. 4a where the corresponding eigenvalue in the PUM case displays a faster convergence rate than the others on the same energy level.

For the Hydrogen atom, in the case of the *hp*-adaptive refinement one observes a superior convergence rate of the first eigenvalue, whereas eigenvalues from the next energy level have errors that are comparable to the *h*-adaptive linear FEM. A possible issue could be related to the smoothness estimation on elements with hanging nodes. In particular it is observed [44] that the smoothness is overestimated when using similar methods, albeit based on Fourier coefficients. This leads to unnecessarily high order polynomial degrees in these areas. Clearly, further investigation is required to resolve this problem.

### Density functional theory

**Fig. 5:**
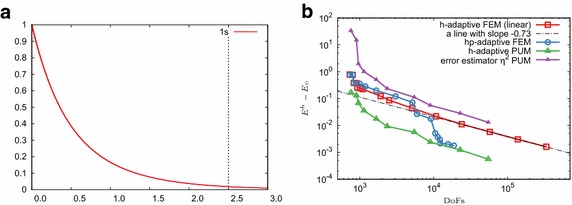
Finite element solution of He atom. **a** Scaled radial solution. The dotted vertical line indicates the enrichment radius. **b** Convergence of the error in total energy of He atom for various FE methods

Finally, we apply the here considered FE approaches to the Kohn–Sham density functional theory. As a first test problem we consider a single He atom which has a single doubly occupied state, i.e. $N_e=2$ and $N=1$. The ground state energy from the radial solution is $E_0=-\,2.834289$. Enrichment functions for PUM are obtained from numerical solution of single atom Schrödinger equations, depicted in Fig. 5a. The atom is placed at the origin in the domain $\Omega =[-\,10;10]^3$ with the homogeneous mesh of size $h=2.5$. Eight elements adjacent to the atom are enriched.

Figure 5b compares the *h*-adaptive FEM, *hp*-adaptive FEM and *h*-adaptive PUM. One immediately recognizes that the PUM leads to a much faster convergence in terms of DoFs and gives about an order of magnitude advantage in terms of the absolute value of the error. The linear *h*-adaptive FEM would require ten times more DoFs to achieve the same accuracy. The *hp*-adaptive FEM displays an exponential-like decay and approaches the accuracy of PUM at higher number of DoFs.

**Fig. 6:**
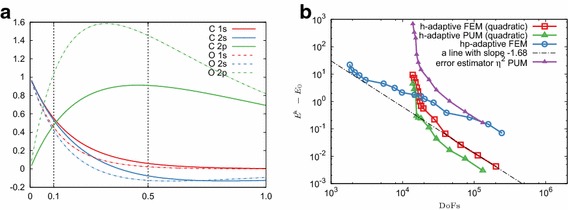
Finite element solution of CO molecule. **a** Scaled radial solution of single atoms. The dotted vertical line at 0.5 indicates the enrichment radius. **b** Convergence of the error in total energy of CO molecule for various FE methods

In the second test problem we consider a CO molecule in the domain $\Omega =[-\,10;10]^3$ at the (equilibrium) distance 2.1. In order to estimate the ground state energy, we fit the total energy obtained by *h*-FEM at the last 3 mesh refinement steps to $\mathrm{ln}(|E^h-E_0|) = C + q\,\mathrm{ln}( \mathrm{DoFs})$ with constraints $C>0,\,E_0<E^h,\,q<0$. Using this approach we estimate the limit of the ground state energy to be $E_0=-\,112.47107$. This renders a bond energy^8^ of $-\,0.5775$, which compares favourably to the value $-\,0.578$ reported in [45]. This gives us confidence to use the estimated ground state energy value in convergence studies, which are presented in Fig. 6.

The enrichment functions for PUM are obtained from the numerical solution of single atom Schrödinger equations; see Appendix B for details. The scaling of those functions are not important for PUM, so Fig. 6a depicts radial solutions normalized so that the value of the 1*s* and 2*s* orbitals are unity at the origin. It is generally possible to use all eigenfunctions from the radial solution as enrichments around each atom in the radius of a few atomic units. However, extra care must be taken not to render the resulting FE space to have linearly dependent basis functions. Figure 6a clearly indicates that given small enough elements (on the order 0.1 a.u.), enriching with both 1*s* and 2*s* single atom radial core electrons solutions would make the FE space degenerate. Our current implementation of PUM DFT only supports enrichment in non-overlapping domains. Therefore for the CO molecule we have to start from a relatively fine mesh, which in the course of *h*-adaptive refinement may render the basis enriched with multiple functions linearly dependent. To avoid this, the PUM results for the CO molecule are obtained by enriching 8 elements adjacent to each atom with its 1*s* orbital only. Scaling of the 1*s* function to unity at the origin of the enrichment spherical function improves the condition number of the resulting matrices.

Figure 6b compares the convergence characteristics using the *h*-adaptive FEM, *hp*-adaptive FEM and *h*-adaptive PUM. The energy error convergence rate from *h*-adaptive FEM compares favourably to the expected rate of $\mathcal {{O}}(h^{2p})$, which can be approximated by $\mathcal {{O}}(\mathrm{DoFs}^{-2p/3})$. Remarkably, the chosen smoothness estimate used in the *hp*-adaptive FEM and its extension to multiple vectors do not lead to an increase in efficiency in terms of the number of DoFs as compared to *h*-adaptive quadratic FEM. The *h*-adaptive PUM displays the same convergence rate as *h*-adaptive FEM and is, as expected, more accurate. This, however, comes at the expense of having a worse condition number for the resulting matrices and the necessity to use higher quadrature order to perform sufficiently accurate numerical integration. For this example and the chosen enrichment radius, the difference in energy error between the two approaches is less than one order of magnitude. By comparing these results to those presented earlier for H and He atoms, we hypothetize that a larger enrichment radius is required to make the PUM advantageous compared to the *h*-adaptive FEM. Our current implementation of PUM DFT, however, only allows enrichment in non-overlapping domains, which limited the enrichment radius for the CO example.

## Conclusions

In this contribution we have applied and critically compared the *h*- and *hp*-adaptive FEM, and the *h*-adaptive PUM to the relevant PDEs in quantum mechanics, namely the Schrödinger equation and the Kohn–Sham all-electron density functional theory. The main findings are summarized below.

- The PUM renders several orders of magnitude more accurate eigenvalues than the standard FEM when solving the Schrödinger equation for the lowest eigenpair with Coulomb and harmonic potential. For the case when more eigenpairs are sought but only the lowest eigenvector is introduced as an enrichment, the PUM is still more accurate, especially for the lowest eigenvalue. Remarkably other eigenvalues also exhibit a faster convergence. The results from DFT calculations indicate that in order to keep this advantage, a reasonably large enrichment radius is needed.
- For problems where a single eigenpair is being sought, the *hp*-adaptive FEM with the here considered smoothness and residual error estimators results in a more accurate solution with fewer number of DoFs as compared to *h*-adaptive PUM and FEM. However, for the case of multiple eigenpairs this approach did not lead to satisfactory results. Overall we find *h*-adaptive PUM to be a more robust solution method to reach the required accuracy even with relatively small enrichment domains.
- Local interpolation error estimates are derived for the PUM enriched with the class of exponential functions. In this case the results are the same as for the standard FEM and thereby admit the usage of the error indicator (7).
- For the PUM DFT calculations the convergence rate of energy error and the residual error estimator are the same for all studied examples. Thus our numerical results confirm that Eq. (7) can be considered as a reliable error indicator for problems in quantum mechanics.
- An element view to the implementation of PUM in FEM codes based on hexahedra is proposed (see Appendix C). As a result, continuity of the enriched field along the edges with hanging nodes is enforced by treating FE spaces produced by each function in the local approximation space separately. The resulting algebraic constraints are independent on the enrichment functions. This allows one to directly reuse algorithms written for enforcing continuity of vector-valued FE spaces constructed from a list of scalar-valued FEs.

## Figures and Tables

**Fig. 7:**
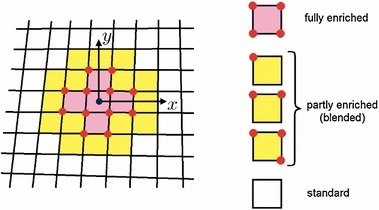
Types of elements in mesh $\mathcal {P}^h$ with respect to the imposed enrichment

**Fig. 8:**
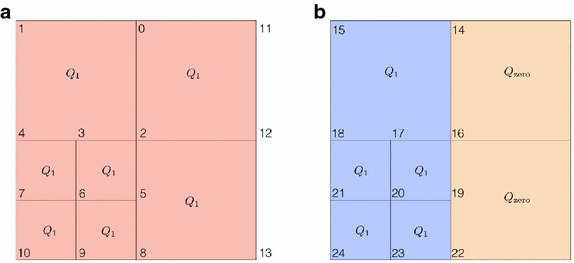
Treatment of hanging nodes for the *h*-adaptive PUM. $Q_1$ denotes (bi)linear FE, whereas $Q_{\mathrm{zero}}$ denotes elements on which functions in the FE space associated with the enrichment function $f_k(\mathbf{x })$ are zero and thus no DoFs need to be introduced. **a** First FE space (standard). **b** Second FE space (enrichment)

**Fig. 9:**
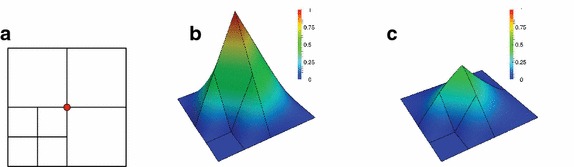
*h* -adaptive mesh refinement and shape functions associated with the central node on the domain $[0,1]^2$ for the standard and enriched element. **a** Mesh. **b** Bilinear. **c** Bilinear enriched with $\exp (-\left|\mathbf{x } \right|)$

## Appendix A: Local interpolation error estimates

In this appendix, the local interpolation error estimates required for the derivation of the error indicator (7) in the case of PUM are obtained for linear finite element approximations enriched with $f(\mathbf{x }) = \exp {(-\mu \,\left|\mathbf{x } \right|^p)}$, where $0 < \mu \in \mathbb {R}$ and $1 \le p \in \mathbb {N}$. These are

12 $$\begin{aligned}&\left\| v-q^h v\right\| _{L^2(K)}\le c_K h_K \left| v\right| _{H^1(\omega _K)}, \end{aligned}$$

13 $$\begin{aligned}&\left\| v-q^h v\right\| _{L^2(e)}\le c_e h_K^\frac{1}{2} \left| v\right| _{H^1(\omega _K)}, \end{aligned}$$

where, as usual, $v:\Omega \rightarrow \mathbb {R}$ is a scalar-valued function, which is assumed to be at least in $H^1(\Omega )$, $q^h$ is a quasi-interpolation operator (of the averaging type), *K* is an element of the discretization $\mathcal {P}^h$ of $\Omega$, $e\subset \partial K$ is an edge of *K*. Also, $h_K$ measures the size of *K*, $\omega _K$ is the patch of elements neighboring *K* including *K* itself. Finally, $c_K,c_e\in \mathbb {R}$ are the interpolation constants independent of the mesh size.

We fix the notations to be used throughout the appendix and make assumptions that are conventional for this kind of analysis. For the sake of simplicity and without loss of generality, we elaborate here for the two-dimensional setting. The obtained results are valid in three dimensions as well.

First, we assume that the partition $\mathcal {P}^h$ of $\Omega \subset \mathbb {R}^2$ consisting of open and convex quadrilaterals *K* is *shape-regular* (or *non-degenerate*), as well as locally *quasi-uniform* in the sense of [46, 47]. For every *K* and its edge *e* we define $h_K:=\mathrm {diam}(K)$ and $h_e:=|e|$ is the length of *e*. For every node *i* in $\mathcal {P}^h$ we denote by $\omega _i$ the union of quadrilaterals connected to node *i* and set $h_{\omega _i}:=\mathrm {diam}(\omega _i)$. Furthermore, for every *K*, $\omega _K$ represents the patch containing *K* and the first row of its neighbors; it is then set $h_{\omega _K}:=\mathrm {diam}(\omega _K)$.

Also, in what follows, by the notation $a\lesssim b$ we imply the existence of a positive constant *C* independent of *a* and *b* such that $a\le Cb$. Then $a\sim b$ means that $a\lesssim b$ and $a\gtrsim b$ hold simultaneously. The symbol $|\cdot |$ will be used to denote either the $H^1$-seminorm (as e.g. in (12) and (13)) or the *length* of a linear segment in $\mathbb {R}^2$ or the *area* of a plane domain in $\mathbb {R}^2$. With these notations at hand, one can show that $|K|^\frac{1}{2}\sim h_K$, $|\omega _i|^\frac{1}{2}\sim h_{\omega _i}$ and $|\omega _K|^\frac{1}{2}\sim h_{\omega _K}$. Furthermore, the shape regularity of the mesh $\mathcal {P}^h$ ensures that $h_e\sim h_K$, whereas its local quasi-uniformity implies that $h_K\sim h_{\omega _i}\sim h_{\omega _K}$.

Finally, we also recall useful inequalities, which are

- the Poincaré-type inequality (see e.g. [48]):
  14 $$\begin{aligned} \left\| v-\frac{1}{|\omega |}\int _\omega v\; d{\mathbf{x }}\right\| _{L^2(\omega )}\le \frac{h_\omega }{\pi } \left| v\right| _{H^1(\omega )}, \quad \forall v\in H^1(\omega ), \end{aligned}$$
  where $\omega \subset \mathbb {R}^n$ ($n=2,3$) is a Lipschitz domain and $h_\omega :=\mathrm {diam}(\omega )$;
- the scaled trace inequality (e.g. in [49], Lemma 3.2):
  15 $$\begin{aligned} \left\| v\right\| _{L^2(e)} \lesssim h_e^{-\frac{1}{2}}\left\| v\right\| _{L^2(K)}+h_e^\frac{1}{2}\left| v\right| _{H^1(K)}, \quad \forall v\in H^1(K). \end{aligned}$$

### Quasi-interpolation operator

Herein, we construct an interpolation operator for obtaining the local error estimates (12) and (13).

Let $V:=H^1(\Omega )$ be an admissible space and $V^h$ be its (enriched) finite element counterpart

16 $$\begin{aligned} V^h&:=\left\{ v^h\in C(\Omega ): v^h({\mathbf{x }}):=\sum _{i\in I^\star } a_i N_i({\mathbf{x }}) + f({\mathbf{x }})\sum _{i\in I^\star } b_i N_i({\mathbf{x }}) \right. \nonumber \\&\quad \left. + \sum _{i\in I^\mathrm {std.}} c_i N_i({\mathbf{x }}), \;\;a_i,b_i,c_i\in \mathbb {R} \right\} \subset V, \end{aligned}$$

where $$I^\star $$ is the set of all enriched nodes of $$\mathcal {P}^h$$ and $$I^\mathrm {std.}$$ is the set of standard, i.e. non-enriched nodes of $$\mathcal {P}^h$$; $$I^\star \cap I^\mathrm {strd.} = \emptyset $$. Recall also that $$ N_i$$ in our case is the $$Q_1$$-shape function associated with node *i* and supported on $$\omega _i$$.

Explicit construction of the operator $q^h:V\rightarrow V^h$ implies the explicit pattern of assignments of $a_i,b_i,c_i\in \mathbb {R}$ through a function $v\in V$. In the case of the enriched FE approximation (16), the major challenge in deriving $q^h$ is imposition of the constant-preserving property on $q^h$, which should be fulfilled on every element $K\in \mathcal {P}^h$ regardless the element type (see Fig. 7).

The operator $q^h:V\rightarrow V^h$ with the desired property reads as follows:

17 $$\begin{aligned} q^h v({\mathbf{x }})&:= \sum _{i\in I^\star } \left[ \frac{1}{2|\omega _i|}\int _{\omega _i} v({\mathbf{y }}) d{\mathbf{y }}\right] N_i({\mathbf{x }}) +\, f({\mathbf{x }})\sum _{i\in I^\star } \left[ \frac{1}{2f({\mathbf{x }}_i)|\omega _i|}\int _{\omega _i} v({\mathbf{y }}) d{\mathbf{y }}\right] N_i({\mathbf{x }}) \nonumber \\&\qquad +\sum _{i\in I^\mathrm {strd.}} \left[ \frac{1}{|\omega _i|}\int _{\omega _i} v({\mathbf{y }}) d{\mathbf{y }}\right] N_i({\mathbf{x }}), \end{aligned}$$

with all notations as in (16) and where ${\mathbf{x }}_i$, entering the second term, denotes the coordinate of a node *i*. Below, for the proposed quasi-interpolation operator of the averaging type $q^h$ we establish that $q^h c|{_K}=c$ on a standard element (note this is a classical result for a non-enriched FEM) and, more importantly, that $q^h c|{_K}= c+\mathcal {{O}}(h_K^p)$ on a fully-enriched and a blended element.

### Estimates

#### Preliminaries

The three estimates that we start with are basic for the following local interpolation error analysis. On every $K\in \mathcal {P}^k$ and its node *i* it holds that

18 $$\begin{aligned}&\left\| N_i({\mathbf{x }}) \right\| _{L^2(K)} \lesssim h_K, \quad \left\| N_i({\mathbf{x }}) \right\| _{L^2(e)} \lesssim h_K^\frac{1}{2}, \end{aligned}$$

19 $$\begin{aligned}&\left| \frac{1}{|\omega _i|}\int _{\omega _i} v({\mathbf{y }}) d{\mathbf{y }} \right| \lesssim h_K^{-1}\left\| v \right\| _{L^2(\omega _K)}+\left| v\right| _{H^1(\omega _K)}, \end{aligned}$$

and

20 $$\begin{aligned} \frac{f({\mathbf{x }})}{f({\mathbf{x }}_i)}= 1+\mathcal {{O}}(h_K^p). \end{aligned}$$

Results (18) rigorously follow from the isoparametric concept and related properties, see e.g. [50] for details. We note that they may be also derived in a less rigorous manner owing to a boundedness of the basis function $$ N_i$$ on *K* along with $$|K|^\frac{1}{2}\sim h_K$$ and $$|e|^\frac{1}{2}=h_e^\frac{1}{2}\sim h_K^\frac{1}{2}$$.

The inequality (19) is obtained as follows:

$$\begin{aligned} \left| \frac{1}{|\omega _i|}\int _{\omega _i} v({\mathbf{y }}) d{\mathbf{y }} \right|&\le |\omega _i|^{-1} \int _{\omega _i} \left| v({\mathbf{y }}) \right| d{\mathbf{y }} \le |\omega _i|^{-\frac{1}{2}} \left\| v \right\| _{L^2(\omega _i)} \\&\lesssim h_K^{-1}\left\| v \right\| _{L^2(\omega _K)}+\left| v\right| _{H^1(\omega _K)}. \end{aligned}$$

Here we used the Cauchy–Schwarz inequality, $|\omega _i|^\frac{1}{2}\sim h_{\omega _i} \sim h_K$ and also the extension-related result $\left\| v \right\| _{L^2(\omega _i)}\le \left\| v \right\| _{L^2(\omega _K)}$.

Finally, to show (20) we explicitly use the properties of $f(\mathbf{x })$. For any fixed *K*, ${\mathbf{x }}\in K$ and ${\mathbf{x }}_i\in K$ being one of its nodes, we have the following upper bound estimate:

21 $$\begin{aligned} \frac{f({\mathbf{x }})}{f({\mathbf{x }}_i)}&= \frac{\exp (\mu |\mathbf{x }_i|^p)}{\exp (\mu |\mathbf{x }|^p)} \le \frac{\exp \left( \mu \left[ \max _{\mathbf{x }\in \overline{K}}|\mathbf{x }|\right] ^p \right) }{\exp \left( \mu \left[ \min _{\mathbf{x }\in \overline{K}}|\mathbf{x }|\right] ^p \right) } \le \frac{\exp \left( \mu \left[ \min _{\mathbf{x }\in \overline{K}}|\mathbf{x }|+h_K\right] ^p \right) }{\exp \left( \mu \left[ \min _{\mathbf{x }\in \overline{K}}|\mathbf{x }|\right] ^p \right) }\nonumber \\&= 1+\mu p\left[ \min _{\mathbf{x }\in \overline{K}}|\mathbf{x }| \right] ^{p-1}h_K + \mathrm {h.o.t.\; in\;} \left\{ \min _{\mathbf{x }\in \overline{K}}|\mathbf{x }|,h_K \right\} \nonumber \\&= 1+\mathcal {{O}}\left( \left[ \min _{\mathbf{x }\in \overline{K}}|\mathbf{x }| \right] ^{p-1}h_K \right) . \end{aligned}$$

Notice that due to boundedness of $\min _{\mathbf{x }\in \overline{K}}|\mathbf{x }|$ for a given fixed *K*, there always exists $\epsilon >0$ such that $\min _{\mathbf{x }\in \overline{K}}|\mathbf{x }|=\epsilon h_K$. Using this in (21), we obtain

$$\begin{aligned} \left[ \min _{\mathbf{x }\in \overline{K}}|\mathbf{x }| \right] ^{p-1}h_K = \epsilon ^{p-1}h_K^p, \end{aligned}$$

yielding, as a result, $\frac{f({\mathbf{x }})}{f({\mathbf{x }}_i)}\le 1+\mathcal {{O}}(h_K^p)$.

The lower bound estimate can be found similarly:

$$\begin{aligned} \frac{f({\mathbf{x }})}{f({\mathbf{x }}_i)}&= \frac{\exp (\mu |\mathbf{x }_i|^p)}{\exp (\mu |\mathbf{x }|^p)} \ge \frac{\exp \left( \mu \left[ \min _{\mathbf{x }\in \overline{K}}|\mathbf{x }|\right] ^p \right) }{\exp \left( \mu \left[ \max _{\mathbf{x }\in \overline{K}}|\mathbf{x }|\right] ^p \right) } \ge \frac{\exp \left( \mu \left[ \min _{\mathbf{x }\in \overline{K}}|\mathbf{x }|\right] ^p \right) }{\exp \left( \mu \left[ \min _{\mathbf{x }\in \overline{K}}|\mathbf{x }|+h_K\right] ^p \right) } \\&= 1-\mu p\left[ \min _{\mathbf{x }\in \overline{K}}|\mathbf{x }| \right] ^{p-1}h_K + \mathrm {h.o.t.\; in\;} \left\{ \min _{\mathbf{x }\in \overline{K}}|\mathbf{x }|,h_K \right\} \\&= 1+\mathcal {{O}}\left( \left[ \min _{\mathbf{x }\in \overline{K}}|\mathbf{x }| \right] ^{p-1}h_K \right) , \end{aligned}$$

and, eventually, $\frac{f({\mathbf{x }})}{f({\mathbf{x }}_i)}\ge 1+\mathcal {{O}}(h_K^p)$. The result (20) then follows.

#### Stability of $q^h$ in $L^2$-norm

The next step towards (12) and (13) implies obtaining the so-called stability result for the constructed $q^h$. Using (18)–(20) one straightforwardly shows that

22 $$\begin{aligned} \left\| q^h v \right\| _{L^2(K)} \lesssim \left\| v \right\| _{L^2(\omega _K)}+h_K\left| v\right| _{H^1(\omega _K)}, \end{aligned}$$

and

23 $$\begin{aligned} \left\| q^h v \right\| _{L^2(e)} \lesssim h_K^{-\frac{1}{2}} \left\| v \right\| _{L^2(\omega _K)} + h_K^\frac{1}{2} \left| v\right| _{H^1(\omega _K)}. \end{aligned}$$

These estimates indeed hold for every *K* regardless of its type (standard, blended, enriched). Note that for a standard non-enriched FEM and the resulting interpolation operators, the estimates (22) and (23) are classical. We have obtained and proved them for our specific operator $q^h$ adopted for the current enriched FEM setting.

#### Constant-preserving property of $q^h$

The final ingredient required for obtaining (12) and (13) is the determination of how “well” the constructed $q^h$ reproduces the constant on an element *K*, depending on its type. This constant-preserving property of the operator is of major importance particularly in the case of enriched FEM.

The required result on a standard (non-enriched) element *K* follows immediately. Indeed, in this case

$$\begin{aligned} q^h v({\mathbf{x }})|_K= \sum _{i=1}^{4} \left[ \frac{1}{|\omega _i|}\int _{\omega _i} v({\mathbf{y }}) d{\mathbf{y }}\right] N_i({\mathbf{x }}), \end{aligned}$$

and the partition of unity $\sum _{i=1}^{4} N_i({\mathbf{x }})=1$ on *K* yields $q^h c|_K=c$, $c=\mathrm {const}$.

The situation on a fully-enriched and partly-enriched (blended) element is more delicate. In the case of a fully enriched element we have

$$\begin{aligned} q^h v({\mathbf{x }})|_K=&\sum _{i=1}^{4} \left[ \frac{1}{2|\omega _i|}\int _{\omega _i} v({\mathbf{y }}) d{\mathbf{y }}\right] N_i({\mathbf{x }}) \\&\quad +\,f({\mathbf{x }})\sum _{i=1}^{4} \left[ \frac{1}{2f({\mathbf{x }}_i)|\omega _i|}\int _{\omega _i} v({\mathbf{y }}) d{\mathbf{y }}\right] N_i({\mathbf{x }}), \end{aligned}$$

that, owing to (20), results in

$$\begin{aligned} q^h c|_K= \frac{1}{2}c + \frac{1}{2}c \sum _{i=1}^{4} \frac{f({\mathbf{x }})}{f({\mathbf{x }}_i)} N_i({\mathbf{x }}) = \frac{1}{2}c + \frac{1}{2}c\left[ 1+\mathcal {{O}}(h_K^p)\right] \sum _{i=1}^{4} N_i({\mathbf{x }}) =c+\mathcal {{O}}(h_K^p). \end{aligned}$$

Now, let *K* be a blended element, implying the representation:

$$\begin{aligned} q^h v({\mathbf{x }})|_K&= \sum _{i=1}^{\ell } \left[ \frac{1}{2|\omega _i|}\int _{\omega _i} v({\mathbf{y }}) d{\mathbf{y }}\right] N_i({\mathbf{x }}) \\&\quad +\, f({\mathbf{x }})\sum _{i=1}^{\ell } \left[ \frac{1}{2f({\mathbf{x }}_i)|\omega _i|}\int _{\omega _i} v({\mathbf{y }}) d{\mathbf{y }}\right] N_i({\mathbf{x }}) \\&\quad +\sum _{i=\ell +1}^{4} \left[ \frac{1}{|\omega _i|}\int _{\omega _i} v({\mathbf{y }}) d{\mathbf{y }}\right] N_i({\mathbf{x }}), \end{aligned}$$

where $\ell \in \{1,2,3\}$ is the number of enriched nodes of *K*. Adding and subtracting the first sum in the above expression, enables us to rewrite it as follows:

$$\begin{aligned} q^h v({\mathbf{x }})|_K&= -\sum _{i=1}^{\ell } \left[ \frac{1}{2|\omega _i|}\int _{\omega _i} v({\mathbf{y }}) d{\mathbf{y }}\right] N_i({\mathbf{x }}) \\&\quad + f({\mathbf{x }})\sum _{i=1}^{\ell } \left[ \frac{1}{2f({\mathbf{x }}_i)|\omega _i|}\int _{\omega _i} v({\mathbf{y }}) d{\mathbf{y }}\right] N_i({\mathbf{x }})\\&\quad +\sum _{i=1}^{4} \left[ \frac{1}{|\omega _i|}\int _{\omega _i} v({\mathbf{y }}) d{\mathbf{y }}\right] N_i({\mathbf{x }}). \end{aligned}$$

Note that the last term contains the summation over all four nodes and is the standard (non-enriched) FE contribution which will automatically reproduce a constant. We then need to estimate, in this context, the remaining part constituting of the first and the second sums. We obtain,

$$\begin{aligned} q^h c|_K&=\frac{1}{2}\,c \sum _{i=1}^{\ell } \left[ \frac{f({\mathbf{x }})}{f({\mathbf{x }}_i)}-1\right] N_i({\mathbf{x }}) +c \le \frac{1}{2}\,c \sum _{i=1}^{\ell } \left| \frac{f({\mathbf{x }})}{f({\mathbf{x }}_i)}-1 \right| N_i({\mathbf{x }}) +c \\&\le \frac{1}{2}\,c \sum _{i=1}^{4} \left|\frac{f({\mathbf{x }})}{f({\mathbf{x }}_i)}-1 \right| N_i({\mathbf{x }}) +c = \frac{1}{2}\,c \,\mathcal {{O}}(h_K^p) \sum _{i=1}^{4} N_i({\mathbf{x }}) +c =c+\mathcal {{O}}(h_K^p), \end{aligned}$$

where (20) was also used.

#### Proof of local error estimates (12), (13)

The derivation of the estimates for $\left\| v-q^h v\right\| _{L^2(K)}$ and $\left\| v-q^h v\right\| _{L^2(e)}$ is based on a combined use of the above stability results for $q^h$, the Poincaré and the scaled trace inequalities (14) and (15), respectively, as well as the constant-preserving property results. First, due to linearity of $q^h$, we have

24 $$\begin{aligned} \left\| v - q^h v \right\| _{L^2(\sigma )}&= \left\| v - c - q^h(v-c) + c-q^h c \right\| _{L^2(\sigma )} \nonumber \\&\le \left\| v - c \right\| _{L^2(\sigma )} + \left\| q^h(v-c) \right\| _{L^2(\sigma )} + \left\| c-q^h c \right\| _{L^2(\sigma )}, \end{aligned}$$

where $c=\mathrm {const}$ and where, for the sake of brevity, we set $\sigma =\{K,e\}$. We are now in a position to dissect every term in (24) in either case of $\sigma$.

When $$\sigma ={{K}}$$ in (24):

By the Poincaré inequality (14), it holds that

25 $$\begin{aligned} \left\| v - c \right\| _{L^2(K)} \le \left\| v - c \right\| _{L^2(\omega _K)} \lesssim h_K \left| v\right| _{H^1(\omega _K)}, \end{aligned}$$

where one can choose $c=|\omega _K|^{-1}\int _{\omega _K} v {\mathrm{d}}{\mathbf{x }}$ and use $h_{\omega _K}\sim h_K$.

By the stability estimate (22) and the Poincaré inequality, it holds similarly to the above that

26 $$\begin{aligned} \left\| q^h(v-c) \right\| _{L^2(K)} \lesssim \left\| v-c \right\| _{L^2(\omega _K)} +h_K \left| v-c \right| _{H^1(\omega _K)} \lesssim h_K \left| v\right| _{H^1(\omega _K)}. \end{aligned}$$

Furthermore, using the results of “Constant-preserving property of $q^h$” section we obtain

27 $$\begin{aligned} \left\| c-q^h c \right\| _{L^2(K)}\equiv 0, \quad \text{ if }\; K\; \text{ is } \text{ standard }, \end{aligned}$$

and

28 $$\begin{aligned} \left\| c-q^h c \right\| _{L^2(K)}= \mathcal {{O}}(h_K^{p+1}), \quad \text{ if }\; K\; \text{ is } \text{ fully } \text{ enriched } \text{ or } \text{ blended }. \end{aligned}$$

In the former case we also use that $\left\| 1 \right\| _{L^2(K)}=|K|^\frac{1}{2}\sim h_K$.

Using (25)–(28) in (24), the resulting local interpolation error of type (12) follows. Note that in the case of fully enriched and blended elements the term $\mathcal {{O}}(h_K^{p+1})$ that appears in the corresponding upper bound can be neglected, being the higher order term with respect to the leading one $h_K \left| v\right| _{H^1(\omega _K)}$.

When $$\sigma ={{e}}$$ in (24):

By the scaled trace inequality (15), it holds

29 $$\begin{aligned} \left\| v - c \right\| _{L^2(e)} \lesssim h_e^{-\frac{1}{2}}\left\| v-c\right\| _{L^2(K)}+h_e^\frac{1}{2}\left| v-c\right| _{H^1(K)} \lesssim h_K^\frac{1}{2} \left| v\right| _{H^1(\omega _K)}, \end{aligned}$$

where we also use $h_e\sim h_K$ along with result in (25).

By the stability estimate (23) and the Poincaré inequality (14), we obtain the result that

30 $$\begin{aligned} \left\| q^h(v-c) \right\| _{L^2(e)} \lesssim h_K^{-\frac{1}{2}} \left\| v-c \right\| _{L^2(\omega _K)} +h_K^\frac{1}{2} \left| v-c \right| _{H^1(\omega _K)} \lesssim h_K^\frac{1}{2} \left| v\right| _{H^1(\omega _K)}. \end{aligned}$$

Finally, using the results of “Constant-preserving property of $q^h$” section we derive

31 $$\begin{aligned} \left\| c-q^h c \right\| _{L^2(e)}\equiv 0, \quad \text{ if }\; K\; \text{ is } \text{ standard }, \end{aligned}$$

and

32 $$\begin{aligned} \left\| c-q^h c \right\| _{L^2(K)}= \mathcal {{O}}(h_K^{p+\frac{1}{2}}), \quad \text{ if }\; K\; \text{ is } \text{ fully } \text{ enriched } \text{ or } \text{ blended }. \end{aligned}$$

In the former case we also use the fact that $\left\| 1 \right\| _{L^2(e)}=|e|^\frac{1}{2}=h_e^\frac{1}{2}\sim h_K^\frac{1}{2}$.

Using (29)–(32) in (24), the resulting local interpolation error estimate of type (13) follows as well. Again, in the case of fully enriched and blended elements the term $\mathcal {{O}}(h_K^\frac{3}{2})$ that appears in the corresponding upper bound can be neglected, being the higher order term with respect to the leading one $h_K^\frac{1}{2} \left| v\right| _{H^1(\omega _K)}$.

## Appendix B: Single atom radial solution

The radial solution of a single atom with charge *Z* is obtained by solving the following coupled problem

33 $$\begin{aligned} \frac{1}{r^2}\frac{\mathrm{d}}{\mathrm{d r}}\left( r^2 \frac{\mathrm{d}}{\mathrm{d r}}\right) R^\phi&= 4\pi \,\rho \end{aligned}$$

34 $$\begin{aligned} \left[ -\frac{1}{2}\frac{1}{r^2}\frac{\mathrm{d}}{\mathrm{d r}}\left( r^2 \frac{\mathrm{d}}{\mathrm{d r}}\right) + \frac{\left[ l+1\right] l}{2 r^2} + V(\rho ,r) \right] R^\psi _{nl}&= \epsilon _{nl} R^\psi _{nl} \end{aligned}$$

35 $$\begin{aligned} \rho&= \sum _n \sum _l \frac{2l+1}{4\pi } f_{nl} {R^\psi _{nl}}^2 \end{aligned}$$

where *n* and *l* are the main and azimuthal quantum numbers, $f_{nl}$ are occupation numbers, *V* is the effective potential and $R^\psi _{rl}$ and $R^\phi$ are respectively radial components of the eigenfunctions and electrostatic potential. The radial component of the eigenfunctions are normalized by $\int _0^\infty r^2 (R^\psi _{nl})^2 \mathrm{dr} = 1$. On the change of variables $U^\psi _{nl} := r\,R^\psi _{nl}$ and $U^\phi := r\, R^\phi$, the system takes the form

36 $$\begin{aligned} \frac{\mathrm{d^2}}{\mathrm{dr^2}} U^\phi&= 4 \pi \,r\, \rho \end{aligned}$$

37 $$\begin{aligned} \left[ -\frac{1}{2}\frac{\mathrm{d^2}}{\mathrm{d r^2}} + \frac{\left[ l+1\right] l}{2 r^2} + V(\rho ,r) \right] U^\psi _{nl}&= \epsilon _{nl} U^\psi _{nl} \end{aligned}$$

38 $$\begin{aligned} \rho&= \sum _n \sum _l \frac{2l+1}{4\pi r^2} f_{nl} {U^\psi _{nl}}^2 \end{aligned}$$

which together with the following Dirichlet boundary conditions

39 $$\begin{aligned} U^\psi _{nl}(0)&= 0 \end{aligned}$$

40 $$\begin{aligned} U^\psi _{nl}(\infty )&= 0 \end{aligned}$$

41 $$\begin{aligned} U^\phi (0)&= 0 \end{aligned}$$

42 $$\begin{aligned} U^\phi (\infty )&= Z \end{aligned}$$

can be solved using the standard finite element method in a sufficiently large but finite domain. The eigenfunctions are then given by $\psi _{nlm} = R^\psi _{nl} Y_{lm}$, where $Y_{lm}$ are spherical harmonics.

## Appendic C: PUM implementational details

An enriched finite element class has been implemented for the general purpose object-oriented C++ finite element library deal.II [30]. The implementation is based on the FESystem class, which is used to build finite elements for vector valued problems from a list of base (scalar) elements. What differs from that class is that the developed FE implementation is scalar, but built from a collection of base elements and enrichment functions^9^

43 $$\begin{aligned} u(\mathbf{x }) = \sum _{i \in I} N_i(\mathbf{x }) u_i +\sum _{k \in S} f_k(\mathbf{x }) \left[ \sum _{j \in I^{\mathrm{pum}}_k} N_{jk}(\mathbf{x }) \widetilde{u}_{jk} \right] , \end{aligned}$$

where *I* is the set of all DoFs with standard shape functions (see Fig. 8a), $I^{\mathrm{pum}}_k$ is the set of all DoFs corresponding to shape functions enriched with $f_k(\mathbf{x })$ (see Fig. 8b) and *S* is the set of enrichment functions.

As distribution of DoFs in deal.II is element based, we always enrich all DoFs on the element. To restore $C^0$ continuity between enriched and non-enriched elements, additional algebraic constraints are added to force DoFs $\widetilde{u}_{jk}$ associated with $N_{jk} f_k$ on the face between the enriched and non-enriched elements to be zero. This is equivalent to enriching only those shape functions whose support is contained within the enriched elements.

The *h*-refinement in deal.II is implemented using hanging nodes. In this case, extra algebraic constraints have to be added to make the resulting field conforming. We build these constraints separately for the non-enriched FE shape functions and enriched shape functions; that is, the following spaces are separately made conforming: $\{ N_i(\mathbf{x })\}$, $\{N_{j0}(\mathbf{x })\}$, $\{N_{j1}(\mathbf{x })\}$, etc. To illustrate this idea consider two separate FE spaces shown in Fig. 8. We assume that functions in the first space are non-zero everywhere in the domain, whereas functions in the second space are non-zero only in the left part, marked by the blue shading. Therefore we do not have to introduce any DoFs in the right part, the underlying elements are denoted by $Q_{\mathrm{zero}}$. The standard procedure implemented in deal.II [27] will enforce continuity of the vector field by introducing algebraic constraints for DoFs associated with hanging nodes^10^ (3, 5, 17, 19), plus constraints for DoFs 14, 16, 22 to make functions in the second FE space zero at the interface between $Q_1$ and $Q_{\mathrm{zero}}$. We can observe now that if we take the constrained scalar field from the first FE space and add a scalar field from the second FE space multiplied by the enrichment functions $f(\mathbf{x })$ (continuous in space), the resulting scalar FE field will also be continuous. Thus we arrive at a conforming *h*-adaptive PUM space where only some elements are enriched. With reference to Fig. 8, the resulting PUM field will have enrichment associated with DoFs 23, 24, 20, 21, 17, 18, 15 whereas DoFs 22, 19, 16, 14, 17 will be constrained.

In this procedure the algebraic constraints do not depend on the enrichment functions and are equivalent to those one would have for the vector-value bases build upon the same list of scalar FEs. Therefore, no extension of the existing functionality to build algebraic constraints was necessary. This allows us to reuse the code written for the FESystem class, which can be used in deal.II to build a vector-valued FE from a collection of scalar-valued elements. Another remarkable benefit of this approach is that existing code can be used to transfer the solution during *h* -adaptive refinement from a coarse to a fine mesh. The reason is that prolongation matrices for enriched elements are equal to their vector-valued counterparts under the condition that all child elements are also enriched. Yet another advantage of implementing a dedicated enriched finite element in deal.II library relates to the numerical integration of jump terms in the Kelly error indicator (see “Error estimator” section). Here, care needs to be taken in computing contributions to cell errors from faces with hanging nodes. Had the authors pursued an implementation of PUM where values and gradients of additional basis functions are evaluated manually via the product rule in the course of numerical integration, a completely separate function would have to be implemented to integrate the jump terms in error indicators, which is not a straight-forward task. We believe that the implementation outlined above is general enough to allow PUM to be applied using deal.II to other partial differential equations such as crack propagation in continuum mechanics. Figure 9 depicts an example of enriched and non-enriched shape functions for the case of *h*-adaptive refinement with hanging nodes in two dimensions.

## Abbreviations

$$\mathbf{x }$$: position vector
$$\lambda _{\alpha }$$: eigenvalue
$$\psi _{\alpha }(\mathbf{x })$$: eigenvector
$$V(\mathbf{x })$$: potential
$$\Omega $$: Lipschitz domain
$$\mathcal {P}^h$$: triangulation of $$\Omega $$
$V^h$: the finite element space of fixed polynomial degree
$\lambda _{\alpha }^h$: eigenvalue obtained from the finite element solution of the problem
$\psi _{\alpha }^h(\mathbf{x })$: eigenvector obtained from the finite element solution of the problem
$\psi ^i_\alpha$: standard degrees-of-freedom
$N_i(\mathbf{x })$: finite element shape functions
$$f_j(\mathbf{x })$$: enrichment functions
$\widetilde{\psi }^{ij}_\alpha$: enriched degrees-of-freedom
$\eta$: explicitly computable error upper-bound
$\eta _K$: local error indicator on the element *K*
$\eta _{K,\alpha }$: local error indicator for eigenpair $\alpha$ on the element *K*
$$a_{ijk}$$: Legendre coefficients
$\sigma _K$: the decay coefficient
$h_e$: the face diameter
$p_K$: the element’s polynomial degree
$p_e$: the maximum polynomial degree over two elements *K* and $K'$ adjacent to the face *e*

## Footnotes

1 Note that in the non-periodic case the Schrödinger equation is actually set in $\mathbb {R}^3$. Therefore the domain $\Omega$ in Eq. (1) is assumed to be sufficiently large such that zero Dirichlet boundary conditions make sense and there is no additional error due to considering a bounded domain. For all example systems that are considered below, the eigenfunctions are known to have asymptotic exponential decay which allows one to choose moderately sized domains.

2 For spin unpolarized systems with an even number of electrons $f_\alpha \equiv 2$ and $N=N_e/2$.

3 We use standard notation for Sobolev spaces and norms.

4 That is, $\eta$ depends solely on the FE solution.

5 That is the measure of how well it is representable by power series.

6 Note that in [22] the Legendre coefficients were required to have even slower decay rate of $\sigma _0 = 0.69$.

7 For spherically symmetric potentials one can separate eigenfunctions into radial $R_{n,l}(r)$ and angular $Y_{m,l}(\theta ,\phi )$ parts, where the latter are spherical harmonics [31]. Here $\{n,l,m\}$ are three quantum numbers.

8 Single-atom energies of C and O atoms are $-\,37.42426$ and $-\,74.46933$, respectively.
